# Nonwoven Mats Based on Segmented Biopolyurethanes Filled with MWCNT Prepared by Solution Blow Spinning

**DOI:** 10.3390/polym14194175

**Published:** 2022-10-05

**Authors:** Pablo Ramos, Tamara Calvo-Correas, Arantxa Eceiza, Javier González-Benito

**Affiliations:** 1 Dept. Materials Science and Engineering, Universidad Carlos III de Madrid, IQMAAB, Av. Universidad 30, 28911 Madrid, Spain; 2 Materials + Technologies’ Group, Faculty of Engineering of Gipuzkoa, Department of Chemical and Environmental Engineering, University of the Basque Country (UPV/EHU), Pza. Europa 1, 20018 Donostia-San Sebastian, Spain

**Keywords:** polyurethanes, solution blow spinning, dielectrics, carbon nanotubes

## Abstract

To prepare nonwoven mats constituted by submicrometric fibers of thermally responsive biopolyurethanes (TPU) modified with multiwalled carbon nanotubes (MWCNT), solution blow spinning (SBS) was used. The TPU was the product of synthesis using poly(butylene sebacate)diol, PBSD, ethyl ester L-lysine diisocyanate (LDI), and 1,3-propanediol (PD) (PBSe:LDI:PD) as reactants. TPU was modified by adding different amounts of MWCNT (0, 0.5, 1, 2, and 3 wt.%). The effect of the presence and amount of MWCNT on the morphology and structure of the materials was studied using field-emission scanning electron microscopy (FESEM) and Fourier-transform infrared spectroscopy (FTIR), respectively, while their influence on the thermal and electric behaviors was studied using differential scanning calorimetry (DSC) and capacitance measurements, respectively. The addition of MWCNT by SBS induced morphological changes in the fibrous materials, affecting the relative amount and size of submicrometric fibers and, therefore, the porosity. As the MWCNT content increased, the diameter of the fibers increased and their relative amount with respect to all morphological microfeatures increased, leading to a more compact microstructure with lower porosity. The highly porous fibrous morphology of TPU-based materials achieved by SBS allowed turning a hydrophilic material to a highly hydrophobic one. Percolation of MWCNT was attained between 2 and 3 wt.%, affecting not only the electric properties of the materials but also their thermal behavior.

## 1. Introduction

Research on materials with low Young’s modulus and especial electric properties is recently receiving great attention because they are highly promising in the field of stretch sensors [1]. When these materials are stretched, at least one electrical property changes, which can then be measured with respect to the strain of the material. Although there are several possible applications, those focused on monitoring different parts of the human body are currently more than relevant [2]. In addition to having the expected electrical performance, these materials must be easily adapted to the surface of the body; thus, they should be soft and lightweight. Accordingly, dielectric elastomer sensors (DESs) seem to be the best candidates. 

On the other hand, the design and preparation of elastic materials with relatively high dielectric constants to fabricate capacitors with intricate shapes or elastic devices for energy harvesting are other interesting issues to consider. For all these applications, room-temperature elastomers seem to be the best choice and, among them, thermoplastics are recommended because they can usually be more easily processed.

Among the most promising elastomers, thermoplastic polyurethanes (TPU) are receiving special attention because they can fulfill the requirements of elasticity, ease of processing, and price [3]. For example, Zahid et al. studied nanocomposites based on thermoplastic polyurethane and reduced graphene oxide to increase electromagnetic interference [4]. Ning et al. prepared blends based on carboxylated nitrile rubber and thermoplastic polyurethane with macroscopic homogeneous high-performance dielectric properties [5]. Ke et al. reported on a strategy to tune the dielectric constant and electric loss of thermoplastic polyurethane composites by using hybrids of carbon nanostructures and graphene nanoplatelets [6]. Furthermore, in the last decades, interest in designing polyurethanes derived from renewable sources has increased because of economic, environmental, and social concerns [7,8,9], since TPUs, in general, have many fields of applications, such as in coatings, adhesives, thermoplastic elastomers, and composites. TPUs are generally constituted by a soft segment (SS; high-molecular-weight polyester or polyether macrodiol) and a hard segment (HS; diisocyanate and low-molecular-weight diol or diamine chain extender). From a micro- or nanostructural point of view, the most important characteristic in these kinds of systems is probably the phase separation occurring because of incompatibility between hard and soft segments. In principle, the extension of phase separation, as well as its associated morphology, can greatly influence the final properties of the TPU. Furthermore, this phase separation depends on copolymer composition, length, hydrogen bonding, and crystallization extent among others [10,11,12,13,14,15]; in turn, these characteristics may be influenced by the processing method used, addition of fillers, and temperature. Therefore, understanding the relationship among structure, morphology, processing, and properties is essential when properly designing materials to tune properties focused on specific applications.

In most cases, polyurethanes are dielectrics with low permittivity and little interest in terms of electrical applications. However, PTUs conveniently modified with conductive particles, such as carbon nanotubes (CNT), may lead to elastomers with special electric properties. These kinds of systems are especially interesting since previous reports indicated that polyether- and polyester-based polyurethanes allow quite uniformly dispersing even nonfunctionalized carbon nanotubes [16,17,18]. Due to their large aspect ratio, high conductivity, high elastic modulus, excellent mechanical properties, and chemical stability, carbon nanotubes offer many advantages when they are used as fillers in polymer composites [19]. For instance, due to their high aspect ratio, they can lead to low electrical percolation thresholds, and they can act as effective nano-reinforcements in materials with improved or tailored mechanical when used at low concentrations [20,21,22,23]. In the case of using thermoplastic polyurethanes as polymer matrices, Barick et al. [23] showed that the conductivity increases with increasing nanotube loading, and the tensile strength and modulus improved in comparison with the neat TPU. Moreover, with the possibility of using elastomeric TPU at room temperature, more applications are available. For example, Shin et al. fabricated a type of conductive sensor based on polyurethane filled with CNTs which could strain up to 300% but with a low gauge factor, ranging from 0.34 to 1.07 [24].

There exist different methods to fabricate CNT/TPU composites, e.g., in situ polymerization of monomers in the presence of CNTs [25], melt mixing process [26], casting CNT/polymer dispersions to prepare films [27], ball mixing and compression molding [28], direct mixing of polymer melt/solution with the CNT powders [29,30,31,32], and mixing of CNT and polymer in solvent and, after its evaporation, processing of composite pellets in a screw extruder [3].

From a technological point of view, it would be interesting to prepare this kind of materials using processes that allow applying them in situ and, therefore, with even the ability of restoration. Additionally, incorporation of a high amount of CNTs into polymers increases the stiffness of the resulting composites and decreases their stretchability [33], which can be inhibitory when highly flexible and soft materials are needed. Therefore, methods to prepare these materials aimed at reducing this stiffening effect are necessary for certain applications. Although some approaches have been tried, such as making perforations using a mechanical punching system [34], they do not ensure in situ material preparation.

Solution blow spinning (SBS) [35,36,37,38] is a relatively recent method that, due to its characteristics, might satisfy all the abovementioned requirements. Furthermore, SBS allows producing materials with morphologies highly affected by the processing conditions, being possible to obtain materials ranging from nonwoven mats (constituted by small fibers with submicrometric diameters) to continuous smooth films. Using this method, a polymer solution (or even a suspension of particles in a polymer solution) is ejected from a concentric nozzle to a certain collector. During the time of flight of the solution the solvent evaporates, with the particles typically being uniformly trapped within the polymer [39,40,41]. Therefore, conveniently choosing the SBS processing conditions enables fabricating highly porous nanocomposites composed of submicrometric fibers, which should favor stretchability. Preparation by SBS of polyurethane composites incorporating multiwalled carbon nanotubes was already reported by Kuk et al. [42]. However, they only confirmed the possibility of obtaining good dispersion of MWNT and gave some data about mechanical properties and thermal stability. Nevertheless, other SBS conditions and TPUs with different chemical structures should be investigated. Furthermore, they did not go deeper into the characterization, e.g., in terms of structure. Lastly, at least simple electrical tests should be conducted if the potential use, e.g., in sensors and capacitors, is expected for these materials. 

Among the available TPUs, thermally activated shape memory polymers offer new challenges in terms of preparing multifunctional materials. If they are filled with conductive nanoparticles such as nanotubes, in addition to tailoring electrical properties, their ability to control shape by temperature should enable new functions for more specific applications. Recently, Calvo-Correas et al. synthesized and characterized microphase separated segmented thermoplastic polyurethanes with different mechanical properties and a wide range of transition temperatures, presenting quite controllable thermally activated shape memory [43]. They are based on a reaction involving macrodiol poly(butylene sebacate)diol arising from castor oil (PBSe), ethyl ester L-lysine diisocyanate (LDI), and 1,3-propanediol as a chain extender (PD).

In the present work, nonwoven mats made of a thermally responsive thermoplastic biopolyurethane modified with multiwalled carbon nanotubes (MWCNTs) were prepared by solution blow spinning (SBS). The effect of the presence and the concentration of MWCNT on the morphology and structure of these materials were studied in order to understand their influence on the thermal, wettability, and electric behavior in terms of capacitance.

## 2. Materials and Methods

### 2.1. Materials

As a biobased thermoplastic polyurethane (TPU) polymer matrix, synthesized by the GMT group of the University of the Basque Country, UPV/EHU was used (M_n_ = 100,000 g·mol^−1^ and polydispersity index, Γ = 2.5) [43]. This polyurethane is based on the reaction mixture formed by poly(butylene sebacate)diol derived from castor oil (PBSe; hydroxyl index of 32.01 mg KOH·g^−1^, and a number-average molecular weight of 3505 g·mol^−1^) [15], ethyl ester L-lysine diisocyanate (LDI; supplied by CHEMOS GmBH), and corn sugar-based 1,3-propanediol (PD; supplied by Quimidroga S.A.), in a molar ratio PBSe:LDI:PD of 1:5:4, corresponding to a 29 wt.% LDI/PD segment content (calculated as weight percentage of LDI and PD with respect to total biopolyurethane weight) and 75 wt.% biobased carbon content. On the other hand, multiwalled carbon nanotubes (MWCNTs) supplied by Sigma-Aldrich were used (more than 95 wt.% purity, 97.9% carbon, diameters ranging from 6 to 9 nm, density of 2.1 g·cm^−3^, and length of 5 μm); the specification sheet of the product does not indicate any functionalization. Tetrahydrofuran (THF; 99.9 wt.%) and acetone (99.9 wt.%) from Sigma-Aldrich were mixed in a proportion of 3:2 *v*/*v* to prepare the solvent to be used in the solution blow spinning process. 

### 2.2. Material Preparation

Before SBS processing, it is necessary to prepare a polymer solution with some characteristics (viscosity, boiling point, surface tension, etc.) favoring fiber formation under the effect of the ejecting gas [44]. Therefore, a preliminary study about TPU solubility and SBS conditions was carried out to ensure the production of fibers. 

Although THF was demonstrated to be one of the best solvents of polyurethanes, it was decided to mix it with the maximum amount possible of acetone, a volatile solvent, to decrease as much as possible the harmfulness of the final solvent. Thus, 40% (*v*/*v*) acetone was the limit composition found at room temperature (24 °C) to ensure dissolution of TPU when the composition of the polymer solution was 10% (*w/v*; dissolving 1 g of polymer in 10 mL of solvent). To prepare the systems to be blow-spun, 1 g of TPU was first dissolved in 6 mL of THF by magnetic stirring, and then, depending on the sample to be prepared, either 4 mL of acetone or 4 mL of a suspension of MWCNT in acetone was added. When preparing suspensions in the TPU solutions, an initial suspension of MWCNT in 4 mL of just acetone was prepared, before subjecting it to a sonication process in an ultrasonic bath for 30 min (this step was carried out to help a previous disaggregation of nanotubes), and then this suspension was added to the TPU solution to achieve the final suspension to be blow-spun. The suspensions were prepared in such a way that, at the end of the process, after the solvent evaporation, materials with compositions 0.0%, 0.5%, 1.0%, 2.0%, and 3.0% by weight of MWCNT were obtained. All preparations were made at 24 °C. The prepared materials were designated as x% MWCNT, where x is the wt.% of MWCNT. 

**Figure 1:**
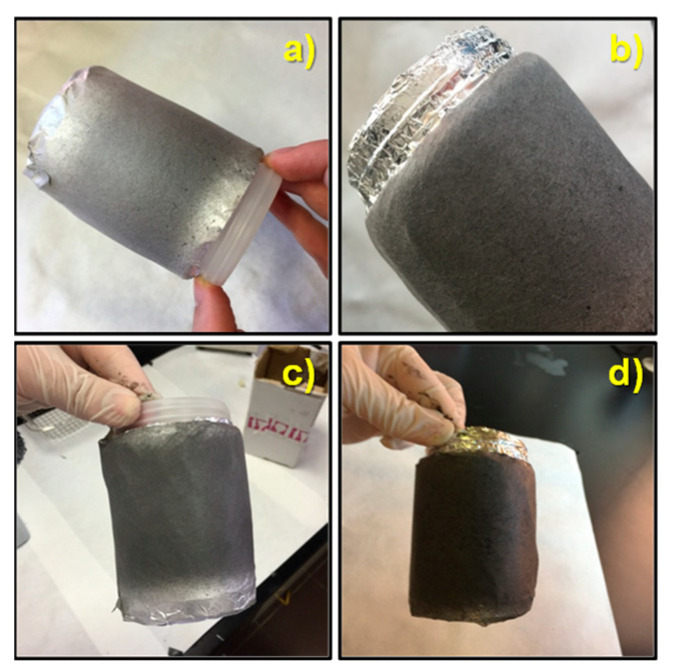
Images of solution blow-spun PBSe:LDI:PD polyurethane-based nanocomposites on the collector: (**a**) 0.5%; (**b**) 1.0%; (**c**) 2.0%; (**d**) 3.0% by weight of MWCNT.

The solution blow spinning was carried out using a machine designed and made in our laboratory [45] inspired by one patented by Medeiros et al. [35,36]. The SBS machine was composed by a nozzle connected to an air compressor with a pressure regulator and a plastic syringe coupled to an automatic pump (NE 1000 X, New Era System, Inc., Farmingdale, NY, USA). The nozzle was made from a cylinder of aluminum perforated along the long axis (diameter 1 mm) to introduce a glass capillary (inner and outer diameters of 0.5 and 0.7 mm, respectively) positioned along the central position with the help of a silicon stopper and protruding 2 mm from the nozzle exit. The aluminum cylinder was also perpendicularly perforated to introduce air at 0.5 bars. With the help of the pump, 10 mL of the mixtures (solution and suspensions) were injected with the syringe at 0.25 mL·min^−1^ in the nozzle to subsequently be ejected at 24 °C via the action of the pressurized air, before finally being deposited on a rotating cylindrical collector wrapped with aluminum foil located at a 15 cm working distance. Figure 1 shows representative images of the materials deposited on the collector.

### 2.3. Characterization Techniques

The morphology of the solution blow-spun samples was studied using field-emission scanning electron microscopy (FESEM) with a TENEO field-emission scanning electron microscope (FEI). The acceleration voltage was set at 1.5 or 2 kV, and the images were obtained from the signal arising from (a) secondary electrons detected using an Everhart Thornley detector (ETD), and (b) backscattered electrons collected using a T1 detector operating in the composite mode A + B. In order to improve the conductivity of the samples, they were carbon-coated by vapor deposition using a Leica EM ACE200 coater. The morphology of the samples and the porosity were studied by image analysis using the free software Image J (NIH, Bethesda, MD, USA). Furthermore, porosity was also determined in terms of the volume of air, *V_air_*, occluded within the samples as the ratio between volume of air respect to the total volume of the sample, *V_s_*.

(1) $$V_{air}=V_{s}-V_{B}=V_{s}-\frac{\omega_{s}}{\delta_{B}},$$

(2) $$Porosity=P=\frac{V_{air}}{V_{s}}=1-\frac{\omega_{s}}{V_{s}\cdot \delta_{B}},$$

where *V_s_* is the volume occupied by the sample, and *V_B_* is the bulk volume or the real volume occupied by the material. On the other hand, *ω_s_* and *δ_B_* are the sample weight and bulk density, respectively. Samples were cut with circular shape, and *V_s_* was determined as

(3) $$V_{s}=A_{s}\cdot d=\left(\frac{1}{4}\pi D^{2}\right)\cdot d,$$

where *A_s_*, *d*, and *D* are the surface area, thickness, and diameter of the sample, respectively. Thicknesses of the tested specimens were measured with a micrometer Digimatic micrometer (Mitutoyo Corporation, Kawasaki, Japan) with an accuracy of ±1 µm and diameters with a conventional caliper. Moreover, specimens were weighted out using a balance with an accuracy of 0.1 mg. Lastly, bulk density was determined using a pycnometer (Micromeritics AccuPyc 1330, Neurtek, Eibar, Spain) obtaining a value of 1.1575 ± 0.0013 (g·cm^−3^). For each sample, at least three specimens were used to measure size parameters and densities to finally give results in terms of the corresponding averages.

The influence of the SBS processing and presence of MWCNT on the TPU structure was studied using Fourier-transform infrared spectroscopy (FTIR) with an FTIR Spectrum GX spectrometer (Perkin-Elmer, Waltham, MA, USA). Spectra were obtained in the transmission mode with the samples directly deposited by SBS over IR transparent KBr discs and averaging interferograms obtained from 20 scans. Spectra were recorded in the range 400 to 4000 cm^−1^ with a resolution of 4 cm^−1^.

Thermal behavior was studied using differential scanning calorimetry (DSC) with a Mettler Toledo 822e. Around 5 mg of sample was weighed out in 50 μL aluminum pans that were subsequently sealed and perforated. Heating and cooling scans were carried out under nitrogen atmosphere. The thermal program used consisted of (i) a first heating scan from −100 to 120 °C at 20 °C·min^−1^ to study the thermal behavior of samples as they were directly obtained from the SBS process, (ii) isothermal treatment at 120 °C for 5 min to ensure full erasing of processing history, and (iii) cooling scan from 120 to −100 °C at 20 °C·min^−1^ to study, during cooling, the effect of the presence of MWCNT. Thermal transitions considered were (a) glass transitions, characterized by the glass transition temperature, T_g_ (determined from the inflexion point when a heat capacity change of the sample takes place), and (b) melting and crystallization characterized by melting temperature, T_m_ (extracted from the peak associated to the endothermic transition observed in the DSC trace obtained during the heating scan) and the crystallization temperature, T_c_ (obtained from the peak associated to the exothermic transition observed in the DSC trace obtained during the cooling scan). Lastly, the crystallinity fraction was determined from the following expression:

(4) $$X_{c}=\frac{\Delta H_{m}}{\left(1-\phi_{MWCNT}\right)\Delta H_{t}},$$

where Δ*H_m_* is the enthalpy of fusion obtained from the area of the endothermic peak obtained arising from the heating scan. Δ*H_t_* is the enthalpy of fusion when a 100% crystalline polymer is considered, defined as Δ*H_t_ =* Δ*H_PBSe_·ϕ_PBSe_*, where Δ*H_PBSe_* is the enthalpy of fusion for the macrodiol, Δ*H_PBSe_*= 142 (J·g^−1^) [43], and *ϕ_PBSe_* is the weight fraction of the macrodiol in the polymer under consideration, *ϕ_PBSe_* = 0.71.

Wettability behavior was studied by contact angle measurements using the sessile drop method with an OCA-15 KRÜSS GmbH tensiometer. Distilled and deionized water was used as testing liquid taking per sample the average contact angle from at least 10 measurements of drops of 3 μL at room temperature, 1 s after their deposition over the samples surface at 1 μL/s.

Lastly, the dielectric measurements were carried out at room temperature with a simple digital multimeter Electro DH Mod.: 60.131. Ensuring good contact, circular specimens of 16 mm were located in between two circular-plane aluminum electrodes (diameter 12 mm) of a home-designed measurement cell connected to the multimeter [1]. Pressure exerted to the specimens in the cell was always the same thanks to the use of a torque wrench (1.18 N·m applied torque). The dielectric constant, *ε*, of the materials was calculated using the simple expression used for a capacitor of parallels plates:

(5) $$\varepsilon =\frac{C\cdot d}{A\cdot \varepsilon_{0}},$$

where *A* is the surface area of the circular plates, *ε_o_* = 8.85 × 10^−12^ F·m^−1^ is the permittivity of vacuum, *C* is the capacitance, and *d* is the thickness of the specimens.

## 3. Results and Discussion

**Figure 2:**
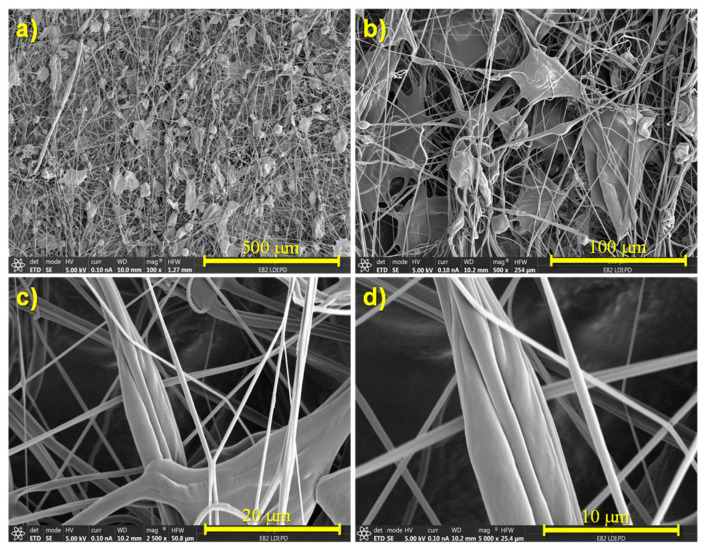
SEM images of the SBS neat TPU, sample without MWCNT, at different magnifications: (**a**) 100×; (**b**) 500×; (**c**) 2500×; (**d**) 5000×.

SEM images at different magnifications from ×100 to ×5000 were used to carry out a morphological study. In Figure 2, as an example, SEM images of the SBS neat TPU sample, without MWCNT, are presented.

**Figure 3:**
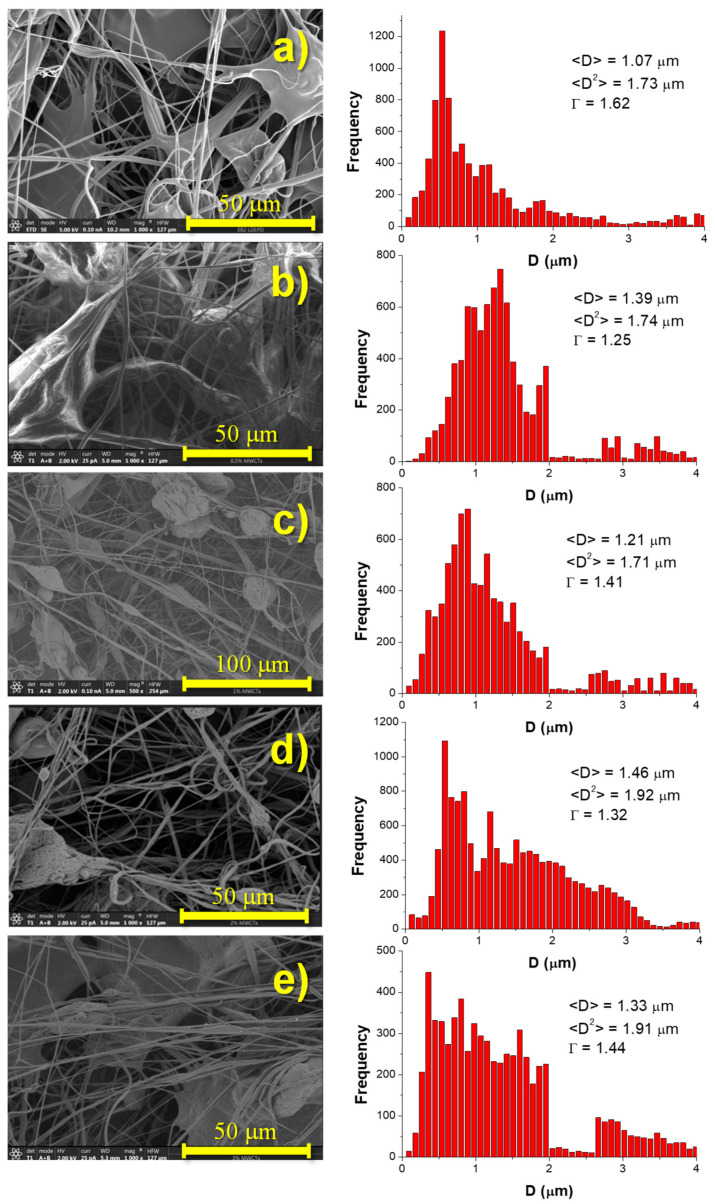
SEM images, diameter distributions, values of average diameter (<*D*>), second moment of the distribution (<*D*_2_>), and dispersion of diameters (*Γ*) of the materials under study: (**a**) 0.0%; (**b**) 0.5%; (**c**) 1.0%; (**d**) 2.0%; (**e**) 3.0% MWCNT.

Regardless of the concentration of MWCNT, the remaining materials prepared in this work showed similar morphologies (Figure 3). The materials mainly constituted fibers of diameters ~1 μm, sometimes interconnected through plane and/or nearly spherical regions where higher material accumulation existed.

In particular, these regions of interconnected fibers constituted fibers that were probably aggregated during the process of fabrication. In addition to isolated fibers and lumps, bundles of fibers could be observed (Figure 2d). When comparing materials with different concentrations of MWCNT (Figure 3), it can be seen that, as the concentration of MWCNT increased, the proportion of fibers with respect to the remaining microstructural features (beads, lumps, etc.) also increased.

Using the software Image J, deeper morphological analysis in terms of diameters of fibers, *D*, and porosity, *P*, was carried out. In Figure 3, SEM images and diameter distributions for every material prepared in this work are presented together with the corresponding values of the average diameter (*<D>*; first moment of the distributions, Equation (6)), the second moment of the distribution (*<D*_2_*>*; Equation (7)), and the dispersion of diameters (*Γ*; given by the quotient between the second and first moments of the distributions, Equation (8)).

(6) $$<D>=\frac{\sum_{i=1}^{n}D_{i}\cdot f_{i}}{\sum_{i=1}^{n}f_{i}},$$

(7) $$<D^{2}>=\frac{\sum_{i=1}^{n}D_{i}^{2}\cdot f_{i}}{\sum_{i=1}^{n}D_{i}\cdot f_{i}},$$

(8) $$\Gamma =\frac{<D^{2}>}{<D>},$$

where *D_i_* is the diameter of a particular fiber *i*, and *fi* is the fraction of fibers with a particular diameter value *D_i_*. 

As can be seen in Figure 3, when adding carbon nanotubes, there was a general tendency of an increase in the size of the regions with material accumulation (in the form of lumps) and a slight increase in the average diameter of the fibers mainly combined with a broadening of the distribution of diameters. Therefore, the presence of MWCNT slightly influenced the morphology, potentially having an important effect on final performance of the materials. 

Changes in the porosity of the materials are among the most important consequences of variations in morphology. The size and relative amount of micro- and submicro-constituents will influence morphology and, therefore, specific properties (properties per unit of mass) and surface properties. Values of porosity were obtained as a mean value arising from at least three measurements on three specimens. In the particular case of neat TPU, the porosity obtained using Equation (2) was *P* = 49% ± 3%, which is quite similar to that obtained using the Image J software (44% ± 1%). This result clearly evidences that morphological analysis through the use of Image J led to information quite close to that obtained from more conventional methods. According to Equation (2), the porosities estimated for the remaining materials were as follows: *P* (0.5% MWCNT) = 52% ± 8%, *P* (1.0% MWCNT) = 45% ± 8%, *P* (2.0% MWCNT) = 45% ± 9%, and *P* (3.0% MWCNT) = 41% ± 10%. Therefore, despite the error of the final values, data indicate a tendency of decreased porosity with the relative increase in MWCNT. It seems, therefore, that there is a relationship between morphological features and porosity. The results obtained suggest that an increase in the proportion of fibers must be the main factor affecting the porosity. More homogeneous microfeatures, such as fibers, may allow better coupling, leading to more compact microstructures with lower porosity.

**Figure 4:**
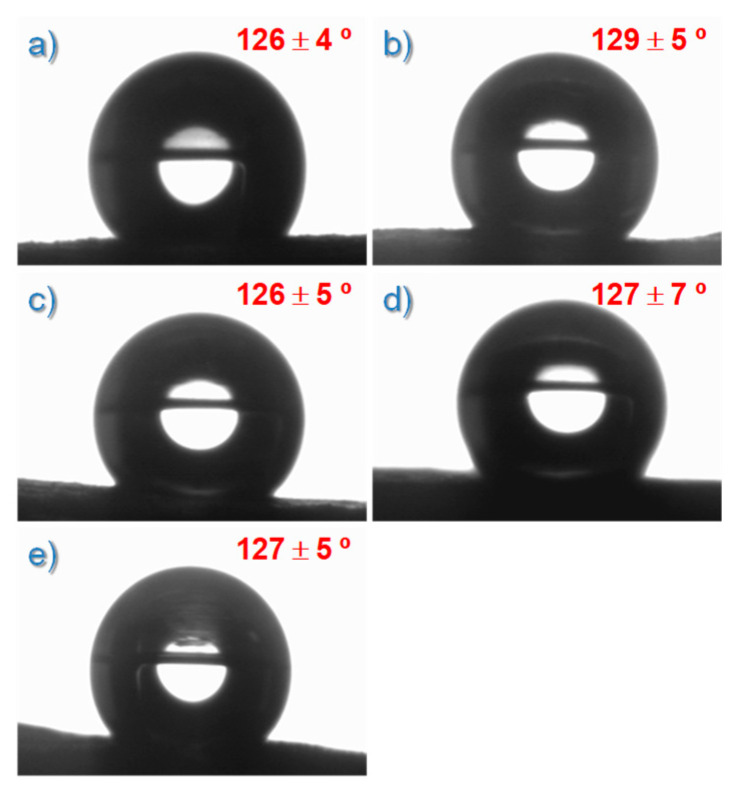
Images of water droplets over the surface of the prepared materials together with the average values of the contact angles obtained: (**a**) 0.0%; (**b**) 0.4%; (c) 1.0%; (**d**) 2.0%; (**e**) 3.0% of MWCNT.

On the other hand, a very important issue to consider, directly affected by the morphology of the materials, is the wettability behavior because of the different environments where the materials under study might work. In order to evaluate the wettability behavior, contact angle measurements were carried out using, as testing liquid, distilled and deionized water. Figure 4 shows representative droplets of water over the surfaces of the materials under study together with the average values of the contact angle obtained. In every case, the materials showed highly hydrophobic behavior with values in the range 126°–129° without any dependence on MWCNT content. Here, it is important to highlight that the water contact angle clearly changed with respect to that obtained for the bulk neat material TPU prepared by hot pressing (78.4° ± 0.4°) [43]. Furthermore, the wettability of carbon nanotubes has been reported to be intermediate since water contact angles are around 90° [46,47]. Therefore, a clearly hydrophilic material (TPU) becomes highly hydrophobic simply by using solution blow spinning as the processing method. It is, therefore, clear that the wettability behavior of these materials is highly affected by the morphology of the specimen tested, in turn induced by the processing method used (SBS).

Two models are commonly used to describe the wettability behavior of rough materials: Wenzel [48] and Cassie–Baxter [49]. In the first, Wenzel defined the relationship between roughness and wettability considering that an increase of surface roughness enhances wettability caused by the chemistry of the surface. Following Wenzel’s model and for the materials under consideration, if the surface is hydrophilic, it would become even more hydrophilic when surface roughness increases. However, our results point out just the opposite. Therefore, the Cassie–Baxter model seems to be more adequate to explain the wettability mechanism of the SBS materials prepared in this work. This approach considers that a higher fraction of air available to the water drop leads to a higher contact angle [49]. Following this reasoning, one would expect that the contact angle changes as a function of porosity. However, the differences in porosity between the materials considered were lower than 22% and possibly even lower when considering the error. Therefore, large changes in contact angle were not expected (Figure 4).

**Figure 5:**
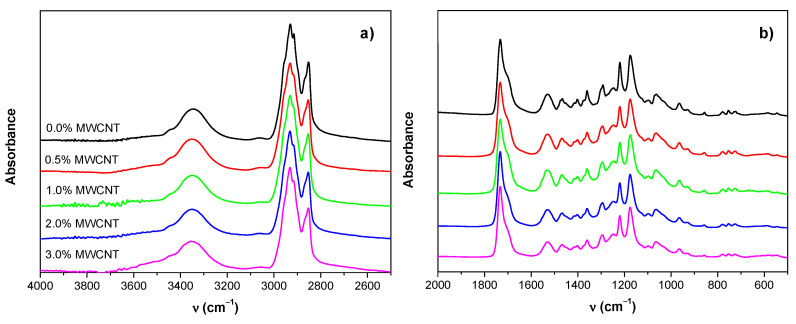
FTIR spectra of all the prepared samples: (**a**) high-energy region; (**b**) low-energy region.

Possible structural changes induced by SBS and the presence of MWCNT were evaluated through the analysis of FTIR spectra. In Figure 5, FTIR spectra of all the prepared materials are presented. To facilitate visualization of the main absorption bands, two regions were chosen, and baseline corrections were applied (Figure 5a,b).

**Figure 6:**
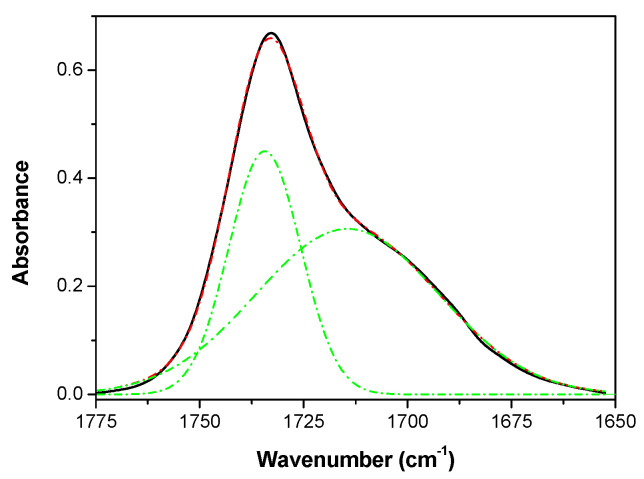
Example of Gaussian deconvolution to obtain the HBI as the ratio of absorption bands A_1703_/A_1732_ (TPU with 2 wt.% MWCNT).

As can be seen, there were no significant differences between the spectra obtained for the blow-spun neat TPU polymer and the spectrum obtained for the hot press neat TPU [43]. Furthermore, as can be seen in Figure 5, all spectra showed peaks located in the same positions, indicating that the incorporation of MWCNT almost did not lead to any structural change. In particular, the typical absorption bands assigned for the same polymer (TPU) in the form of the film obtained by hot pressing [43] were identified. For example, the broad band appearing between 3200 and 3400 cm^−1^ was composed of overlapped absorption bands associated with the N–H stretching vibration (free and hydrogen bonded) of urethane groups. Moreover, peaks at 2929 cm^−1^ and 2854 cm^−1^ with shoulders at 2955 cm^−1^ and 2866 cm^−1^ could be assigned to the C–H stretching vibrations of –CH_2_ and –CH_3_, respectively. On the other hand, the most prominent peak observed at 1732 cm^−1^ with a shoulder at about 1703 cm^−1^ corresponded to the stretching vibrational mode of the free and hydrogen bonded –C=O carbonyl of the urethane linkage (–HN–COO–) [43,50] and the ester group of the PBSe, respectively. Considering that MWCNTs were not modified by oxidation (they were used as received), special adhesion between them and TPU through specific interactions (mainly hydrogen bonds) was not expected. Therefore, any changes in the contribution of the hydrogen-bonded C=O group were mainly due to changes in the degree of phase separation [51] induced by the presence of MWCNT. Hydrogen bonding was expected between the NH groups as the proton donors and the oxygens as proton acceptors in the carbonyls of the LDI/PD segment and in the esters of the macrodiol. The hydrogen bonding index (HBI) can be defined as the ratio of absorption bands A_1703_/A_1732_, where *A_i_* represents the area or absorbance of the band at a wavenumber *i* determined using a Gaussian curve fitting method (Figure 6). Lastly, the degree of phase separation (*DPS*) was obtained using the following equation [52]:

(9) $$DPS=\frac{HBI}{HBI+1}\times 100.$$

The values obtained for the DPS of the different materials prepared were as follows: 64% (0.0% MWCNT), 64% (0.5% MWCNT), 67% (1.0% MWCNT), 63% (2.0% MWCNT), and 63% (3.0% MWCNT). These results indicate that the addition of MWCNT scarcely affected the phase separation. There was a slight increase in phase separation upon adding a small number of nanotubes up to 1%; then, at higher MWCNT content (2% and 3%), the degree of phase separation was recovered. This result may be interpreted considering that, without reaching MWCNT percolation, when the concentration of nanotubes was enough, specific interactions between nanotubes and polymer chain associated with the PBSe could slightly increase the inter- and intramolecular interactions by hydrogen bonds between the LDI/PD segments. However, when percolation was reached between 1% and 3% MWCNT, as reported for other polymers filled with nanotubes [1], less of the nanotube surface was available for the interactions with polymer. 

Analyzing the absorbance ratios, using the most intense band (C=O band) where some variations are expected, it is possible to extract more information about possible interactions between MWCNT and the polymer. For instance, taking the band at 2930 cm^−1^ assigned to the antisymmetric stretching mode of the methylene group –CH_2_, the following evolution of its absorbance ratio with respect to the carbonyl group can be observed: A_2930_/A_1730_, 0.44 (0.0% MWCNT); 0.46 (0.5% MWCNT); 0.46 (1.0% MWCNT); 0.33 (2.0% MWCNT); 0.30 (3.0% MWCNT). Thus, when adding a small number of nanotubes (0.5%, 1.0% MWCNT), the ratio A_2930_/A_1730_ increased slightly compared to the SBS neat polymer but decreased in the samples with the highest content of carbon nanotubes (2.0% and 3.0% MWCNT). This fact could be explained again considering a direct relationship between the available MWCNT surface and specific polymer–nanotube interactions. When the content of MWCNT is low enough, good dispersion with isolated nanotubes will be within the polymer favoring interactions between the surface of the nanotubes and the methylene groups of the polymer chains. However, by increasing the concentration of MWCNT (2.0% and 3.0% MWCNT), interconnections between the nanotubes will occur, favoring aggregate formation, thus reducing specific interactions with the polymer chains, in accordance with the analysis of the evolution of absorption band of hydrogen bonded carbonyl groups. It is also necessary to highlight that, considering the proportion of MWCNT with respect to the polymer, the contribution in the FTIR spectra of the bands associated with C–H stretching modes of the carbon nanotubes should be negligible respect to those arising from the polymer. 

**Figure 7:**
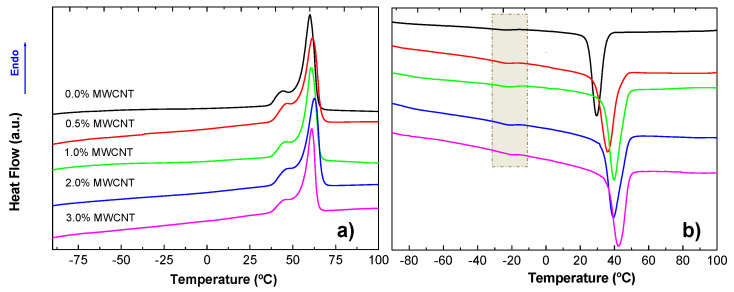
Thermograms corresponding to the (**a**) first heating and (**b**) subsequent cooling DSC scans.

In Figure 7, thermograms corresponding to the first heating and subsequent cooling DSC scans are shown. Throughout the heating (Figure 7a), regardless of the concentration of carbon nanotubes, only a clear endothermic peak, *T_m_*, (61 °C) with a shoulder, *T_shoulder_*, at a lower temperature (45 °C) could be observed. They were associated with the melting temperature of an ordered microdomain mainly formed by PBSe. In the case of semicrystalline polymers, it is usually stated that the melting process depends on the distribution of crystallite sizes, the presence of different crystal forms, domain sizes, and different degrees of order in the crystalline structure [1,53,54]. Therefore, crystalline heterogeneity might be the main reason why the shoulder appeared. 

Furthermore, for temperatures of both the peak and the shoulder (Table 1), there was a slight shift to higher temperatures when MWCNTs were added to the TPU, suggesting that the presence of nanotubes favored the crystallization, probably due to an enhanced preferential chain orientation when phase separation was favored, as already observed for other PU systems filled with modified MWCNTs [55]. In fact, when the concentration of MWCNTs was increased to 3%, the fraction of crystallinity, *X_c_*, increased by 10% (Table 1). On the other hand, the *T_g_* values reported for the same polymer, but prepared by hot pressing, which appeared at −40 °C (assigned to the PBSe-based domain) and 15 °C (assigned to the LDI/PD-based domain) [43], cannot be clearly distinguished in the thermograms of Figure 7a. The main reason for difficulties in the observation of *T_g_* is the high crystallinity degree of the materials (Table 1), whose effect may be, in addition, enhanced by the preferential orientation of the polymer chains when the materials are prepared in the form of fibers due to the SBS process. However, it seems that, when materials are prepared by SBS, less crystallinity is achieved in comparison with similar materials prepared by hot pressing, at least when they are slowly cooled [43].

In the DSC traces of the cooling scans (Figure 7b), the exothermic crystallization peak at higher temperatures can be clearly observed as the amount of MWCNT increased (Table 1). This result indicates the existence of a nucleating effect caused by the presence of carbon nanotubes. On the other hand, at least one *T_g_* (−18 °C) can be observed (selected area in Figure 7b). This result may be explained by considering that, after erasing the processing history (SBS), the extra chain order given by the SBS process disappeared, allowing better visualization of the glass transition temperature. Additionally, the crystallization degree was at least 10% lower because the chosen cooling rate must have been relatively fast for this type of system.

**Figure 8:**
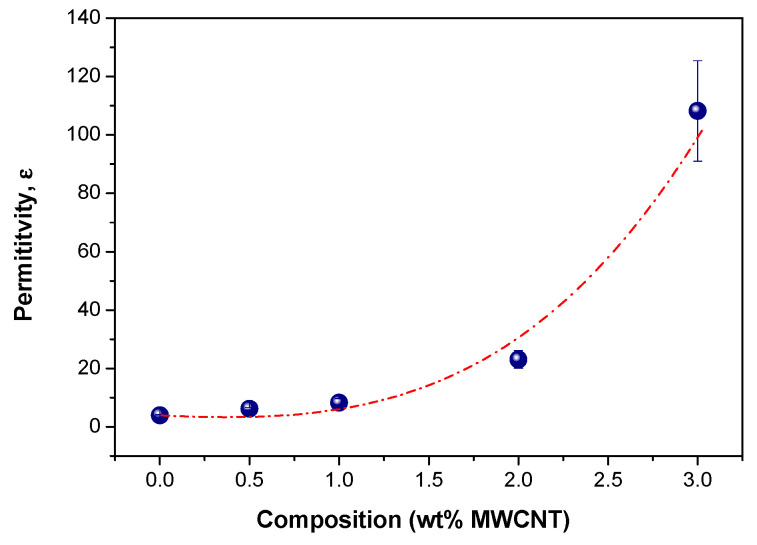
Permittivity (calculated from Equation (5)) as a function of MWCNT content.

Lastly, in order to better visualize the effect of the presence of MWCNT on the electrical behavior, a plot of the permittivity (calculated from Equation (5)) as a function of the carbon nanotube concentration is shown in Figure 8. It can be observed that the permittivity slightly increased at low concentrations of MWCNT up to about 1 wt.%, followed by an abrupt increase at 2 wt.%. This behavior indicates the existence of a clear transition (electrical percolation) in the system from dielectric to conductive material in the range 2–3 wt.%. In fact, values of percolation fraction from 2 to 3 wt.% are commonly found in the literature for many thermoplastic polymers filled with MWCNT [37] and even for TPU filled with MWCNTs [56,57]. Designing materials aimed at controlling the point at which this transition occurs is more than essential, since it would be possible to prepare materials with a wide range of electrical properties ranging from materials having relatively low dielectric constant to materials with a certain conductivity that might even be even tuned with temperature (piroelectricity).

## 4. Conclusions

Solution blow spinning was satisfactorily used to prepare highly porous fibrous materials based on thermally responsive biopolyurethanes modified with multiwalled carbon nanotubes (MWCNTs). When adding carbon nanotubes there was a change in the morphology mainly observed as an increase in the relative number of submicrometric fibers as the amount of MWCNT increased, which was finally reflected by a decrease in porosity.

The Cassie–Baxter approach explained the wettability behavior of the SBS TPU-based materials prepared in this work where hydrophilic material became highly hydrophobic simply by using solution blow spinning as the processing method.

The addition of MWCNT scarcely affected the phase separation in the TPU; however, there was a slight increase in phase separation upon adding a small amount of carbon nanotubes up to 1 wt.%; then, at higher MWCNT content of 2 and 3 wt.%, the degree of phase separation was recovered.

When percolation was reached between 1 and 3 wt.% MWCNT, there were changes not only in the electric properties but also in the thermal behavior. At high concentrations of MWCNT, less of the carbon nanotube surface was available for the polymer chains to interact. When the content of MWCNT was low enough, they were uniformly dispersed within the polymer, favoring interactions between the surface of the nanotubes and the methylene groups of the polymer chains. However, upon increasing the concentration of MWCNTs (2 and 3 wt.%), interconnections between the carbon nanotubes occurred, favoring aggregate formation and, therefore, reducing specific interactions with the polymer chains. 

It was demonstrated that SBS is a convenient method to prepare TPU-based materials with tailored properties with potential applications as non-wettable DESs. Here, it is important to highlight that, using SBS, these materials can be prepared in situ to be perfectly adapted to almost any surface, regardless of its shape.

## Figures and Tables

**Table 1:** Thermal parameters obtained from the first heating and cooling DSC scans of the prepared materials.

%MWCNT	*T_m_* (°C)	*T_shoulder_* (°C)	*X_c_*	*T_c_* (°C)
0.0	60.1	44.4	0.76	29.6
0.5	61.5	46.7	0.80	35.9
1.0	60.8	46.0	0.82	39.9
2.0	62.8	46.7	0.79	39.6
3.0	61.1	46.4	0.88	42.6

## Data Availability

The data of this study are available upon request.
